# Non-Pharmacological Stroke Prevention in Atrial Fibrillation

**DOI:** 10.3390/jcm12175524

**Published:** 2023-08-25

**Authors:** Iñigo Anduaga, Alessandro Affronti, Pedro Cepas-Guillén, Jorge Alcocer, Eduardo Flores-Umanzor, Ander Regueiro, Salvatore Brugaletta, Eduard Quintana, Laura Sanchis, Manel Sabaté, Xavier Freixa

**Affiliations:** 1 Cardiology Department, Institut Clínic Cardiovascular, Hospital Clínic de Barcelona, 08036 Barcelona, Spain; 2 Cardiovascular Surgery, Institut Clínic Cardiovascular, Hospital Clínic de Barcelona, 08036 Barcelona, Spain; 3 Faculty of Medicine and Health Sciences, University of Barcelona, 08007 Barcelona, Spain

**Keywords:** atrial fibrillation, left atrial appendage occlusion, oral anticoagulation, stroke

## Abstract

Atrial fibrillation (AF) is the most common arrhythmia worldwide. It is associated with increased mortality and morbidity, especially due to the increased risk of ischemic stroke and systemic embolism in these patients. For this reason, thromboembolism prevention is the cornerstone of managing AF, and oral anticoagulation is nowadays the first-line treatment. However, since most thrombi form in the left atrial appendage and anticoagulant therapy may have side effects and be contraindicated in some patients, surgical and percutaneous left atrial appendage occlusion (LAAO) have emerged as a non-pharmacological alternative. This review summarizes all existing evidence on surgical and percutaneous LAAO.

## 1. Introduction

Atrial fibrillation (AF) is the most common arrhythmia worldwide. It is estimated to affect 2–4% of the adult population, and its prevalence increases with advancing age [1,2]. Given the aging population, AF is expected to become even more prevalent in the coming years [3]. Ischemic stroke and systemic embolism are the most significant risks associated with AF, leading to increased morbidity and mortality. Therefore, thromboembolism prevention is the cornerstone of managing AF [4,5].

The standard of care for stroke prevention in AF is oral anticoagulation (OAC), which includes both vitamin K antagonists (VKA) and direct anticoagulants (DOAC) [1,6]. However, surgical and percutaneous left atrial appendage occlusion (LAAO) have emerged as non-pharmacological alternatives for stroke prevention in AF patients [1]. Several studies have demonstrated their effectiveness and safety in different contexts, raising their importance in daily clinical practice [7,8,9,10]. This review aims to summarize all existing evidence on surgical and percutaneous LAAO. 

## 2. Rationale for Left Atrial Appendage Occlusion

Systemic embolism is the main risk of AF, cardioembolic stroke being its most-feared presentation. It has been shown that AF is about 20–30% of ischemic strokes and 10% of cryptogenic ones [1]. The risk of cardioembolic stroke in patients with AF is modulated according to age and comorbidities, increasing up to more than 15% per year in older patients with previous cardiovascular comorbidities [11,12].

The relationship between thrombus, systemic embolism, and AF has been known for several decades [13], with the left atrial appendage (LAA) playing a significant role in this pathological process. In 1947, Hellerstein et al. reported on eight LAA resections in dogs, postulating a possible therapeutic role in patients with rheumatic mitral disease [14]. In 1949, Madden et al. already observed the presence of thrombus in the LAA in patients with AF and recurrent systemic arterial embolism and reported LAA exclusion in two patients undergoing mitral commissurotomy [15]. Subsequently, in 1955, Belcher and Somerville noted that 64% of patients who underwent mitral valvotomy and experienced a systemic embolism had thrombi in the LAA, compared to only 16% in those who did not experience an embolic event [16]. Despite these findings, the interest in LAAO remained limited for several decades until 1996, when a systematic review reported that over 90% of thrombi in nonvalvular AF were in the LAA. Numerous studies in the following years confirmed these significant observations [17,18]. 

These findings have helped to support that, since thrombi are formed in the LAA, when closing this structure, we are able to eliminate the source of the origin of cardioembolic events. This fact, along with the presence of patient (high bleeding risk) and systemic (suboptimal OAC complaint) barriers, have fueled the development of endovascular and surgical procedures to exclude the LAA as a non-pharmacologic approach for stroke prevention in AF patients [19].

## 3. Surgical Left Atrial Appendage Occlusion

### 3.1. Clinical Outcomes

Surgical LAAO can be performed either as a stand-alone procedure or, more commonly, as a concomitant procedure during a cardiac operation for other indications, such as valve surgery or coronary bypass grafting (CABG). The former is currently uncommon due to the increased availability of less invasive percutaneous techniques. In contrast, concomitant LAAO is carried out more frequently, and it can be categorized into two different clinical scenarios:

#### 3.1.1. Concomitant LAAO Surgery in Patients with Previous AF

Since the first report in 1948 [15], LAAO has been targeted in patients with a history of AF as a concomitant procedure during other cardiac operations to decrease the risk of embolic stroke. For many years, LAA exclusion was sporadically performed using non-standardized techniques as an adjunct to mitral surgery. Substantial observational evidence has been accumulated in this regard, yielding diverse results but predominantly indicating a positive impact of concomitant LAAO in preventing ischemic stroke following cardiac surgery [20]. However, the evidence was primarily based on case reports or small series with highly variable outcomes. Left atrial appendage occlusion study (LAAOS) trials have demonstrated the effectiveness and safety of LAAO in patients undergoing cardiac surgery, leading to a paradigm shift and impacting clinical practice [21,22,23]. Table 1 provides a summary of their characteristics and results.

The Left Atrial Appendage Occlusion Study I (LAAOS I) trial was the first study to assess the safety and efficacy of LAA occlusion, using sutures or a stapling device, at the time of coronary artery bypass grafting (CABG) [21]. This study showed that a high success rate (87% of complete occlusion of the LAA after cardiac surgery) could be achieved with experience (>4 cases). In the same line, it showed good safety results, with no significant increase of operative time, bleeding, or heart failure. Except for one intraoperative ischemic stroke and one perioperative TIA, no additional strokes were detected after an average of 13 ± 7 months of follow-up.

The Left Atrial Appendage Occlusion Study II (LAAOS II) trial explored the feasibility of LAAO for stroke prevention in AF patients undergoing heart surgery [22]. After performing a cross-sectional study of 1889 consecutive patients undergoing cardiac surgery, which showed a 10.8% AF rate and 5.2% AF and increased stroke risk rate, 51 patients were randomized to LAAO or standard care. No significant differences were observed in the efficacy endpoint (composite of death, myocardial infarction (MI), stroke, noncerebral systemic emboli, or major bleeding), even though stroke was less frequent in the occlusion arm (3.9%) compared to the no occlusion arm (12%). Of note, the rate of patients recruited per center was low (1.6 per center per month).

After the publication of the LAAOS II trial, a meta-analysis summarized all available data on LAAO in patients with AF undergoing cardiac surgery [24]. A total of 3653 patients (1716 patients with concomitant LAAO versus 1937 patients without LAAO during cardiac surgery) were analyzed from two randomized trials and five observational studies. The LAAO group showed a lower incidence of stroke at 30 days (0.95% versus 1.9%; OR 0.46, *p*-value = 0.005) and during follow-up (1.4% versus 4.1%; OR 0.48, *p*-value = 0.01). The LAAO group also exhibited a significantly reduced all-cause mortality (1.9% versus 5%; OR 0.38, *p*-value = 0.0003), with similar rates of postoperative AF and reoperation for bleeding compared to the non-LAAO group. The authors concluded that concomitant LAAO appears to be a promising strategy for reducing the stroke risk in patients with a history of AF, both in the short and long term, without a significant increase in complications. Based on this evidence, the 2017 STS guidelines for atrial fibrillation surgery recommended LAAO during concomitant cardiac operations in patients with previous AF (Class IIa, level C) [25].

The Left Atrial Appendage Occlusion Study III (LAAOS III) trial was designed to overcome the limitations of the previous studies [23]. This multicenter randomized clinical trial assessed the role of LAAO during cardiac surgery in patients with AF and increased risk of stroke (CHA2DS2-VASc score ≥ 2). This trial was superior in the primary endpoint (first occurrence of ischemic stroke or noncerebral systemic embolism after cardiac surgery) in the LAAO group with a larger difference after the first 30 days after surgery. There were no differences in the secondary and safety endpoints, such as all-cause mortality, rehospitalization for heart failure, major bleeding, and myocardial infarction. This landmark and well-powered randomized clinical trial (RCT) provided robust evidence regarding the effectiveness of surgical LAAO during cardiac surgery in patients with AF, specifically in preventing strokes and embolisms. Notably, 76.8% of participants in both groups received OAC at the 3-year follow-up, indicating that surgical LAAO offers additional protection against strokes when combined with OAC therapy. Therefore, we cannot conclude that LAAO during cardiac surgery should replace OAC instead of being seen as a complement. Unlike percutaneous LAAO, this trial did not support using surgical LAAO as a stand-alone alternative to OAC therapy.

#### 3.1.2. Concomitant LAAO Surgery in Patients without Previous AF

The existing evidence regarding the potential embolic protection of concomitant surgical LAAO in patients without a preexisting history of AF remains unclear. Yao et al. analyzed the effect of surgical LAAO on mortality and stroke in a cohort of over 75,000 patients who underwent cardiac surgery. Among them, 25,721 (33.9%) had preexisting AF, and 4374 (5.8%) underwent LAAO. The average follow-up duration was 2.1 years. In the subgroup of patients without a previous AF, concomitant surgical LAAO was not associated with a reduced risk of stroke or mortality [26]. In a study conducted by Melduni et al. from the Mayo Clinic group, the influence of concomitant LAAO during cardiac surgery on the occurrence of perioperative AF, stroke, and all-cause mortality was assessed, involving a propensity score-matched analysis of 9792 patients [27], 54% of them with no history of previous AF. LAA closure was independently associated with an increased risk of early perioperative AF (adjusted OR, 3.88; 95% CI, 2.89–5.20) but did not significantly reduce the risk of stroke (adjusted HR, 1.07; 95% CI, 0.72–1.58) or mortality (adjusted HR, 0.92; 95% confidence interval, 0.75–1.13). 

The AtriClip Left Atrial Appendage Exclusion Concomitant to Structural Heart Procedures (ATLAS) study was a prospective, randomized study that examined the feasibility of LAAO in surgical patients who developed postoperative AF [28,29]. Patients without previous AF but with a high ischemic risk (CHADS2-VASc score ≥ 2) were randomly assigned to two groups: concomitant LAAO with an AtriClip device (*n* = 376) and no LAAO (*n* = 186). The success rate of the LAAO procedure (no flow nor residual stump >10 mm) was 99%. Perioperative AF developed in 47.3% of participants in the LAAO group and 38.2% in the no LAAO group (*p*-value = 0.047). In patients who developed perioperative AF, thromboembolic events were observed in 3.4% of LAAO patients and 5.6% of patients without LAAO (*p*-value = 0.40). Based on the above evidence, surgical LAAO concomitant to cardiac surgery in patients without preexisting AF should not be recommended.

### 3.2. Surgical Techniques and Devices

**Figure 1:**
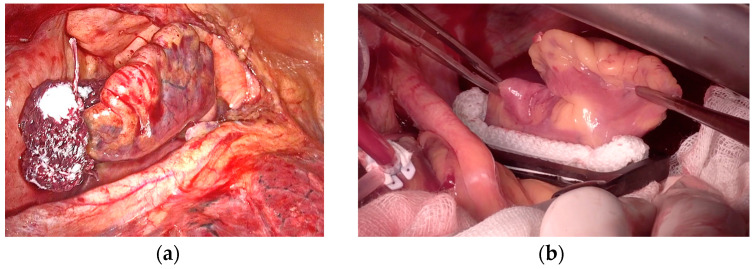
Left atrial appendage surgical exclusion by occlusion technique. (**a**) AtriClip in positioning by left thoracoscopy. (**b**) Measurement and implantation of AtriClip by median sternotomy.

LAA surgical exclusion can be achieved through various methods, which can be broadly categorized into two techniques: excision techniques and occlusion techniques [30,31,32]. Excision techniques involve resecting the LAA and suturing the remaining tissue directly or by means of a stapler. On the other hand, occlusion techniques aim to isolate the LAA from circulation while leaving it in place (Figure 1). The occlusion technique can be subdivided into endocardial direct surgical suture, stapler occlusion without excision, and device-based LAA occlusion. Table 2 provides the main classification and advantages and disadvantages of each technique.

The initial occlusion technique for LAAO was endocardial circular purse string suture, which was later modified due to its inefficiency. They transitioned to a single- or double-layer running suture. However, despite this modification, the suture lines often remain incomplete, leading to LAA thrombosis and an elevated risk of embolism [32,33,34,35]. Katz and colleagues evaluated the efficacy of LAA endocardial ligation in patients undergoing mitral valve surgery, with no positive outcomes [36]. Incomplete exclusion was identified in 36% of the patients who underwent the procedure. Among this group, 50% also exhibited spontaneous echo contrast or thrombi in the LAA, and 22% experienced postoperative thromboembolic events. Similar results were observed in the cohort studied in 2015 by Aryana et al. [37]. These findings suggest that the technique was unsuccessful in completely excluding the left atrial appendage, leading to an increased risk of thrombotic events.

Surgical LAA closure with staplers, with or without stump excision, was then introduced as an approach to address the problem of incomplete closure. However, it was commonly observed that bleeding through the stapler line and recanalization of the lumen occurred in cases where non-excision techniques were employed [38,39]. 

Kanderian et al. retrospectively compared the results of three LAA exclusion techniques: surgical excision, surgical occlusion, and stapling occlusion [32]. The success rate of LAAO was modest: only 55 out of 137 (40%) LAAO were successful, the surgical excision being the most successful technique with only a 73% success rate. As for events, in a retrospective study by Lee et al., surgical excision was associated with a lower risk of stroke or TIA than all other occlusion techniques (*n* = 710, 0.2% versus 1.1%; *p* = 0.001) [40]. Among the study limitations, the low overall incidence of late neurological events and the wide variety of procedures in the “alternative techniques” group should be considered. A small pilot RCT comparing three LAA closure techniques: internal surgical ligation, surgical excision, and stapler excision confirmed the previous discouraging results, as the overall failure rate was 57%—in this case, with no significant differences between the three techniques [41].

Based on the previous results, an editorial by Marc Gillinov concluded that the “standard surgical management of the LAA is unsuccessful in the majority of cases” [42]. The shortcomings of traditional surgical techniques have led to the development of occlusion devices which effectiveness is essentially based on exerting higher and more uniform occlusion pressure than suture occlusion and stapling. The AtriClip (AtriCure, Mason, OH, USA), consisting of two polyester-covered parallel tubes with nitinol springs, is the most studied LAA occlusion device. Its application results in a necrosis line between the closure elements that effectively excludes the LAA from circulation. Other advantages include its rapid deployment and the possibility of reorientation and reapplication. Moreover, the risk of tearing the LAA or causing injury to the circumflex artery is extremely low [43]. Modified device versions have been introduced recently, allowing for minimally invasive or stand-alone total thoracoscopic procedures [44,45]. In the EXCLUDE trial, 61 patients undergoing LAA exclusion with the AtriClip were examined using TEE or CT at three months. The occlusion success rate was 98.4% [46]. Emmert et al. evaluated the long-term results of AtriClip LAA exclusion in 40 patients undergoing elective cardiac surgery. Computed tomography scans at 3, 12, 24, and 36 months showed 100% clip stability with no displacement. No thrombi, LAA perfusion, or LAA stumps were detected. Clinically, no strokes or TIAs were reported [47]. The AtriClip may also exert an anti-arrhythmogenic effect. Starck et al. showed complete electrical isolation of the LAA using AtriClip in 10 patients with AF undergoing off-pump CABG with concomitant bilateral pulmonary vein isolation [48].

Other surgical techniques include epicardial snare loops, LAA invagination and suture, and other variants described only in case reports or small series, the results of which are not generalizable on a larger scale.

### 3.3. Special Considerations in Surgical LAA Management

The management of LAA can potentially address both deleterious consequences attributed to its presence in the setting of AF—namely, thrombus formation and arrhythmia persistence. It is well known that the LAA may play a role in the maintenance of advanced forms of atrial fibrillation. For this, either complete surgical excision or interruption of myocardial perfusion to the LAA (with AtriClip) will ultimately suppress the electrical contribution to AF. From a surgical standpoint, direct excision during concomitant cardiac surgery under cardiopulmonary bypass (CPB) is potentially the most effective technique for achieving embolic risk reduction and eliminating the electrical input source. Data extracted from the LAAOS III trial [23] place the surgical management of the LAA in patients with AF as a clearly advantageous, safe, and simple procedure. The inherent advantage, through surgery, of avoiding intracardiac footprints left with percutaneous occluder devices and data observed in the LAAOS III poses the question of whether more patients could benefit from minimally invasive epicardial LAA obliteration. Thrombi in the LAA constitutes a contraindication for external surgical stapling or clipping on the beating heart. Instead, such findings would favor surgical excision of the entire appendage with direct intracavitary vision and control. 

Beyond the LAA excision, surgical ablation for AF has been successfully utilized for over 3 decades. However, its role and the surgical approach continue to be debated. Regardless of the AF type, a recent report demonstrated that the Cox maze procedure for stand-alone AF is safe and effective [49]. The highest one-time procedural success rate and stroke reduction were found for the Cox maze procedure with CPB compared with any catheter or off-pump surgical ablation procedure [50,51]. Thus, consideration for concomitant or stand-alone AF ablation should be given to fit patients being considered for LAA percutaneous or surgical occlusion. 

Observational data strongly support the wide adoption of the most extensive AF ablation procedure (Biatrial Cox maze intervention) during concomitant cardiac surgery [52,53,54,55,56,57,58,59]. The multilevel benefits of such an intervention in terms of restoration of the sinus rhythm; control of LAA; and potentially secondary benefits (improved hemodynamics, reduced thromboembolic events, improved quality of life, restoration of left ventricular dysfunction, and potentially increased survival) make this procedure a Class I recommendation in the STS guidelines during concomitant cardiac surgery [60]. However, there is still no robust and consistent evidence arising from randomized trials on the utility of this Biatrial maze operation for many of the described hard outcomes [61,62,63,64,65]. 

Regarding the lifelong safety of the most used clipping or occlusion devices, very long-term data on the potential nuances of such hardware left in the intracardiac or in the pericardial space is still pending. The possibility of late erosion into the surrounding structures, late infection, or the potential to complicate cardiac interventions needs to be considered. For this, in very young patients, surgical excision and direct closure at the time of cardiac surgery seem the most appropriate and cost-effective course of action [66].

## 4. Percutaneous Left Atrial Appendage Occlusion

### 4.1. Clinical Outcomes

Randomized data supporting the efficacy of LAAO are limited, with only three published randomized controlled trials (RCT) comparing LAAO to the standard of care (OAC). These trials include PROTECT AF (2009), PREVAIL (2014), and PRAGUE-17 (2020) [67,68,69]. Table 3 provides a summary of their characteristics and results.

The PROTECT AF (Watchman Left Atrial Appendage System for Embolic Protection in Patients With Atrial Fibrillation) trial was the first trial, followed by the PREVAIL (Evaluation of the Watchman LAA Closure Device in Patients With Atrial Fibrillation Versus Long-Term Warfarin Therapy) trial. In both trials, the patients were randomly assigned in a 2:1 ratio to either LAAO using the WATCHMAN device or warfarin. The PROTECT AF trial demonstrated non-inferiority of the primary endpoint (a composite of stroke, systemic embolism, and cardiovascular or unexplained death) in the LAAO group. However, safety concerns emerged due to an increased risk of procedural complications in the LAAO group, particularly cardiac tamponade and procedure-related strokes [67]. Consequently, the PREVAIL trial was conducted to evaluate the safety of the WATCHMAN device. This trial achieved non-inferiority for a secondary coprimary endpoint of postprocedural ischemic stroke. However, it was not achieved for the first composite coprimary endpoint of stroke, systemic embolism, or cardiovascular mortality [68]. It is worth noting that the warfarin arm in the trial had a very low ischemic stroke rate (0.73%), which deviated significantly from previous data. A meta-analysis of patients from the PROTECT AF and PREVAIL studies followed for 5 years demonstrated similar events in both groups for the composite endpoint. However, LAAO was associated with a significant decrease in hemorrhagic stroke (HR 0.2, CI 0.07–0.56, *p* = 0.0022), disabling stroke (HR 0.45, CI 0.21–0.94, *p* = 0.03), and non-procedure-related bleeding (HR 0.48, CI 0.32–0.71, *p* = 0.0003), as well as reduced cardiovascular (HR 0.59, CI 0.37–0.94, *p* = 0.027) and all-cause (HR 0.73, CI 0.54–0.98, *p* = 0.035) mortality compared to OAC [8]. Similar results were observed in the CAP (Continued Access to PROTECT AF) and CAP2 (Continued Access to PREVAIL) registries, which were designed to gather additional data on the safety and efficacy of the WATCHMAN device. With an average follow-up of 50 months, LAAO was associated with a reduction in stroke rates of over 69–78% compared to the predicted stroke rates based on the CHA2DS2-VASc scale [70]. Several important characteristics of these trials should be mentioned. Firstly, patients with contraindications to OAC were excluded. Secondly, an intensive anticoagulation protocol was administered after LAAO, consisting of VKA for 45 days, followed by DAPT for 6 months, and then lifelong aspirin. Notably, the control group in these trials received VKA as OAC treatment, not DOAC.

The third randomized trial, PRAGUE-17 (Left Atrial Appendage Closure vs. Novel Anticoagulation Agents in Atrial Fibrillation), was designed to overcome these limitations. In the PRAGUE-17 trial, 402 patients were randomized to LAAO versus DOAC (with Apixaban being the most frequently administered DOAC). Notably, these patients presented both a high ischemic risk (CHA2DS2-VASc score of 4.7 ± 1.5) and bleeding risk (HAS-BLED score of 3.1 ± 0.9), and the median follow-up duration was 20.8 ± 10.8 months. The primary endpoint was a composite of combined ischemic, bleeding, and procedural events, demonstrating non-inferiority when comparing LAAO with DOAC. The event rate was similar in both groups (sHR 95% CI = 0.84 (0.53–1.31), *p* = 0.004 for non-inferiority), indicating that LAAO was non-inferior to DOAC. Additionally, procedure- or device-related complications occurred in only nine patients (4.5%) [69]. These results were consistent in a subsequent analysis with a follow-up period of 4 years [9].

Several nonrandomized studies have demonstrated the safety and efficacy of LAAO in preventing ischemic stroke and bleeding events. However, it is essential to acknowledge that these studies have various limitations, including heterogenous endpoints, absence of a control arm, potential bias due to their observational design, and conclusions drawn from comparing the ischemic and bleeding risk with the predicted risk using CHA2DS2-VASc and HAS-BLED scores [71]. A recently published propensity score study that included 562,850 patients with atrial fibrillation from large US databases compared patients treated with LAAO (8397 patients) versus those treated with DOAC (554,453 patients). LAAO was associated with no significant difference in the risk of the primary composite endpoint—ischemic stroke or systemic embolism, major bleeding, and all-cause mortality (HR, 0.93 (0.84–1.03))—or the secondary outcomes, including ischemic stroke/systemic embolism (HR, 1.07 (0.81–1.41)) and intracranial bleeding (HR, 1.08 (0.72–1.61)). However, LAAO was associated with a higher risk of major bleeding (HR, 1.22 (1.05–1.42), *p* = 0.01) and a lower risk of mortality (HR, 0.73 (0.64–0.84), *p* < 0.001). The lower risk of mortality associated with LAAO was most pronounced in patients with a prior history of intracranial bleeding [71]. For these reasons, large, randomized clinical trials that compared LAAO versus the standard care (especially with DOAC) are needed to confirm the real impact of this promising therapy.

### 4.2. Safety Outcomes

The most common periprocedural complications associated with LAAO procedures are pericardial tamponade, occurring in approximately 0.29% to 4.3% of cases, and vascular complications, ischemic stroke, or device embolization, which have an incidence of around 1%. Notably, the incidence of pericardial tamponade has decreased over the years, and experienced operators currently report an incidence of approximately 1% [72,73,74]. Table 4 provides an overview of the main periprocedural and postprocedural complications.

**Figure 2:**
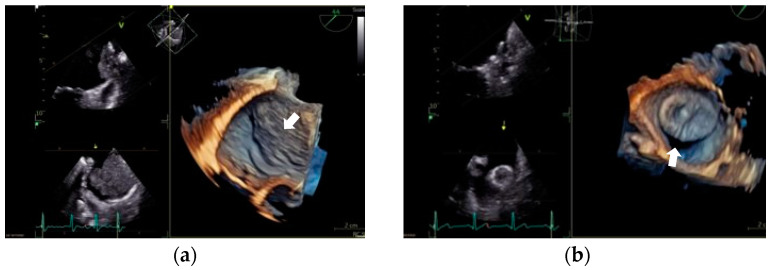
Primary postprocedural complications. (**a**) Device-related thrombus. Transesophageal echocardiogram. Midesophageal plane at 33°. It shows a large thrombus (46 × 35 mm) that covers the entire device and extends along the entire surface of the Marshall ligament. (**b**) Peri-device leak. Transesophageal echocardiogram. Midesophageal plane at 33°. It shows a significant gap between the pulmonary ridge and the device.

The two primary postprocedural complications associated with LAAO are device-related thrombus (DRT) and a peri-device leak (PDL) [75]. A visual representation is provided in Figure 2.

Device-related thrombus (DRT) has an incidence rate ranging from 3% to 7% and has been linked to an increased risk of ischemic stroke and all-cause death after LAAO [76,77]. Several risk factors have been proposed, most of which are nonmodifiable, such as age, previous stroke, hypercoagulability disorders, and renal insufficiency. Given its impact, there is a growing interest in identifying modifiable risk factors to prevent its development. The current evidence has demonstrated that iatrogenic pericardial effusion, deep device implantation, and antithrombotic treatment after LAAO are associated with DRT occurrence. However, the treatment for DRT is not well established, and several strategies have been proposed [74,78,79,80,81].

A peri-device leak (PDL) occurs when complete closure of the LAAO is not achieved. The incidence of PDL has varied across studies due to the lack of consensus in detection and classification. However, it is approximately 26.5% at 45 days for any leak, the incidence of large leaks (>5 mm) approximately 0.7%. [75,82]. Recent studies have associated PDL development with increased stroke and thromboembolic events [82,83,84]. Similar to DRT, there is a lack of evidence regarding the management of PDL. Therefore, treatment should be individualized, and periodic monitoring using a transesophageal echocardiogram, anticoagulation, and leak closure may be considered in some cases [75].

### 4.3. Device Characteristics

**Figure 3:**
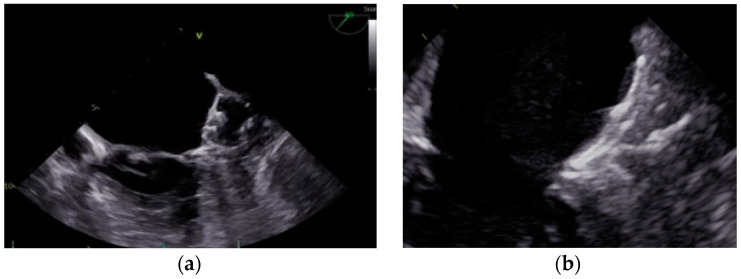
Percutaneous LAAO device categories. (**a**) Plug-based devices. Transesophageal echocardiogram. Midesophageal plane at 49°. Plug-based WATCHMAN FLX™ device. (**b**) Disc-/lobe-based devices. Transesophageal echocardiogram. Midesophageal plane at 73°. Disc-/lobe-based Amplatzer™ Amulet™ device.

The main current catheter-based devices for LAAO can be categorized into two groups. Figure 3 provides a visual representation of these devices.

(a) Plug-based devices: These devices feature a lobe or umbrella that seals the neck of the LAA, preventing blood flow into the LAA. The most used device in this group is the WATCHMAN™ device (Boston Scientific, Marlborough, MA, USA).
(b) Disc-/lobe-based devices: These devices consist of a lobe or umbrella and an additional disc that seals the ostium of the LAA from the left atrial side. The Amplatzer™ Amulet™ device (Abbott Vascular, Santa Clara, CA, USA) is the most widely used in this category.

The WATCHMAN 2.5 device (Boston Scientific) was the second dedicated LAAO device (the first device was the PLAATO device (Appriva Medical), withdrawn from the market in 2006). The WATCHMAN 2.5 device has been replaced by the second generation of the WATCHMAN device, known as WATCHMAN FLX (Boston Scientific). The WATCHMAN FLX device offers several advantages compared to its predecessor:

(a) It features a polyethylene terephthalate full-cover membrane cap, which helps minimize the risk of a peri-device leak (PDL).
(b) The device has a higher number of struts (18) and anchors (12 in two rows), along with increased radial strength, providing greater stability.
(c) It comes in five different sizes, ranging from 20 to 35 mm, allowing for better customization due to patient anatomy.
(d) The WATCHMAN FLX device is fully recapturable and repositionable, with an atraumatic closed distal end, facilitating precise placement during the procedure.

While most of the evidence regarding WATCHMAN devices is based on the initial WATCHMAN 2.5 device, the results with the first-generation WATCHMAN devices differed significantly from those of the second generation.

The PINNACLE FLX (The Protection Against Embolism for Non-valvular AF Subjects: Investigational Evaluation of the WATCHMAN FLX™ LAA Closure Technology) trial was a single-arm study specifically designed to assess the efficacy and safety of the second-generation WATCHMAN FLX device. In this trial, 400 patients were enrolled, and all patients achieved the primary effectiveness endpoint, which involved no peri-device leaks greater than 5 mm at the 12-month follow-up. Two patients experienced the primary safety endpoint, including one case with ischemic stroke and one transient ischemic attack. Notably, no device embolization or pericardial effusion were observed [85]. Similar positive results have been reported by the National Cardiovascular Data Registry (NCDR), which included a large cohort of 16,446 patients treated with the WATCHMAN FLX device [74]. These findings further support the efficacy and safety of the WATCHMAN FLX device in clinical practice.

The Amplatzer Amulet device (Abbott) is the second device approved by the FDA for left atrial appendage occlusion (LAAO). It consists of two components: a lobe and a disc. The dual-seal technology of the Amplatzer Amulet device allows for filling the left atrial appendage (LAA) cavity with the lobe and sealing the ostium with the disc, providing effective closure. The Amplatzer Amulet device is an improvement and evolution of its predecessor, the ACP device. It comes preloaded in eight sizes, ranging from 16 to 34 mm, to accommodate LAA sizes from 11 to 31 mm (based on landing zone measurements). It requires a minimum LAA depth of 12 mm for appropriate placement.

The RCT Amulet IDE Trial, a randomized controlled trial, included 1878 high-risk patients for LAAO and compared the Amplatzer Amulet device to the WATCHMAN 2.5 device. The primary endpoint of the trial was a residual leak at 45 days. The Amplatzer Amulet device demonstrated a significant improvement in the primary endpoint, with no residual leak observed in 63% of patients compared to 46% with the WATCHMAN 2.5 device. However, no significant difference was observed in severe peri-device leaks (>5 mm), which occurred in 1% of the Amplatzer Amulet group and 3% of the WATCHMAN group. Notably, a higher risk of pericardial effusion was observed in the Amplatzer Amulet group: 2.43% versus 1.23%. Similar findings were reported in the SWISS-APERO trial, which compared the Amplatzer Amulet device (111 patients, 50.2%) to the WATCHMAN device (25 patients with WATCHMAN 2.5 and 85 patients with WATCHMAN FLX). No significant difference was observed in the peri-device leaks at follow-up, but a higher risk of periprocedural complications was observed in the Amplatzer Amulet group (9.0% versus 2.7%; *p* = 0.047) [86]. Real-world data from observational studies have also demonstrated the efficacy and safety of the Amplatzer Amulet device in various complex clinical scenarios [87,88].

There are other less widely used devices, and their characteristics are included in Table 5 [89,90,91]. The presentation of previously used devices and devices in development is far from the scope of this review.

## 5. Indications According to Societies and the Recent Consensus

In both the 2020 ESC guidelines and the 2019 AHA/ACC/HRS atrial fibrillation guidelines, percutaneous LAAO is reserved for patients with AF and contraindications to the long-term use of anticoagulants (IIb), while surgical LAAO is considered for patients with AF undergoing cardiac surgery (IIb) [1,6]. The 2023 SCAI/HRS Expert Consensus Statement strongly emphasizes the careful selection of patients for percutaneous LAAO. Patients with a good quality of life and a life expectancy of at least one year should be considered for LAAO. Discussions between the patient and their provider are crucial in making a shared decision [92]. The good results in both the efficacy and safety outcomes due to advances in devices, imaging, and technique and the publication in the coming years of ongoing studies may increase its evidence and indications in the near future [92].

## 6. Future Directions

Several ongoing clinical trials are currently comparing percutaneous LAAO with OAC [93,94]. In patients contraindicated for OAC or with a history of life-threatening bleeding, clinical trials have also compared LAAO with OAC, antiplatelet therapy, or no treatment [95,96]. These studies are summarized in Table 6 and will provide further evidence in the coming years. In surgical LAAO, The Left Atrial Appendage Exclusion for Prophylactic Stroke Reduction (LeAAPS) Trial will definitively answer whether LAAO in patients without preexisting AF undergoing cardiac surgery is safe and effective for stroke prevention (NCT05478304). LeAAPS, currently underway, will randomize 6500 patients without AF but with an increased risk for stroke to concomitant LAAO with AtriClip or not. LeAAPS is powered for the primary outcome of stroke or systemic emboli (unlike the ATLAS study) [30].

## 7. Conclusions

Surgical and percutaneous LAAO have emerged as non-pharmacological treatments for preventing stroke in patients with AF. Surgical LAAO should be considered in AF patients undergoing other cardiac interventions. Device-based LAA occlusion is the most recommended surgical technique at present, although, in very young patients, surgical excision and direct closure should be considered. As for percutaneous LAAO, it is currently indicated in patients with contraindications to the long-term use of anticoagulants. It is expected that, in the following years, as the techniques are refined and new clinical trials are published, percutaneous and surgical LAAO will increase their evidence and expand their indications.

## Figures and Tables

**Table 1:** Characteristics and results of randomized surgical trials.

Trial	Design	Results
LAAOS I (2005)	Surgical LAAO (*n* = 52; suture or stapler) versus standard therapy (*n* = 25)	Complete occlusion LAA: Suture 45% versus Stapler 72%; *p*-value = 0.14 Rate of complete occlusion LAA: initially 43% versus 87% after 4 cases (*p*-value = 0.0001) Perioperative thromboembolic events: Surgical LAAO 3.8% versus Control group 0%; (*p*-value = 1) No additional strokes at follow-up (13 ± 7 months)
LAAOS II (2013)	Surgical LAAO (*n* = 26; ‘cut-and-sew’ technique) versus No LAAO and OAC (*n* = 25)	Efficacy endpoint (1y): Compound of death, MI, stroke, SE, or major bleeding: Surgical LAAO 15.4% versus Control group 20%; (*p*-value = 0.61)
LAAOS III (2020)	Surgical LAAO and ACO (*n* = 2379; suture, stapler, or LAAO device) versus no LAAO and OAC (*n* = 2391)	Stroke or SE (3.8 years): Surgical LAAO 4.8% versus Control group 7%; (*p*-value 0.001) No differences in all-cause mortality, rehospitalization for heart failure, and myocardial infarction (3.8 years)
ATLAS (2022)	LAA exclusion (*n* = 376; AtriClip^®^) versus OAC (*n* = 186)	Success rate (no flow nor residual stump >10 mm) of 99% Perioperative AF (1y): LAA exclusion 47.3% versus Control group 38.2%; (*p*-value = 0.047) Thromboembolic event after POAF (1y): LAA exclusion 3.4% versus Control group 5.6%; (*p*-value = 0.40) Bleeding events (1y): LAA exclusion 23% versus Control group 5.4%; (*p*-value = 0.005) 30-day and 1-year mortality did not show differences (*p* = 0.35, *p* = 0.36)

LAAO: left atrial appendage occlusion; LAA: left atrial appendage; OAC: oral anticoagulation; AF: atrial fibrillation; MI: myocardial infarction; FU: follow-up; SE: systemic embolism; POAF: perioperative AF; RR: risk ratio; CI: confidence interval; HR: hazard ratio.

**Table 2:** Characteristics of surgical LAAO techniques.

Technique	Group	Advantage	Disadvantage
LAA resection and suture	Excision	No risk of thrombi formation (no residual stump)	Time consuming Risk of bleeding
Single /double layer direct suture	Occlusion	Easy to perform Cheap	High rate of incomplete LAA exclusion
Stapler without stump resection	Occlusion	Easy to perform	Risk of recanalization of lumen over time
Stapler with stump resection	Excision	Easy to perform	Risk of bleeding
Occlusion devices (AtriClip)	Occlusion	Easy application Extremely high rate of effective LAA isolation Possibility of reorientation and/or reapplication Possibility of use in minimally-invasive surgery and stand-alone thoracoscopic procedures Possible role in achieving LAA electric isolation	Cost? Risk of LAA laceration or circumflex artery injury/distortion (both very low)

LAAO: left atrial appendage occlusion; LAA: left atrial appendage.

**Table 3:** Characteristics and results of randomized percutaneous trials.

Trial	Design	Results
PROTECT-AF (2009)	LAAO (*n* = 463; Watchman) versus warfarin non-inferiority (*n* = 244)	Stroke, SE, and CV or unexplained death (18 months): LAAO 3/100 patient-years versus Control group 4.9/100 patient-years; RR (95% CI) = 0.62 (0.35–1.25); non-inferiority probability >99.9% Major bleeding, pericardial effusion, and device embolization (18 months): LAAO 7.4/100 patient-years versus Control group 4.4/100 patient-year; RR (95% CI) = 1.69 (1.01–3.19)
PREVAIL (2014)	LAAO (*n* = 138; Watchman) versus warfarin non-inferiority (*n* = 269)	Follow-up 11.8 ± 5.8 months (only 28% reached 18 months) Stroke, SE, and CV or unexplained death: LAAO 0.064 versus Control group 0.063; RR (95% CI) = 1.07 (0.57–1.89) Significant non-inferiority was not achieved (upper limit: 1.89) Stroke or SE after 7 days of randomization: LAAO 0.025 versus Control group: 0.02; Risk difference (95% CI) CI = 0.0053 (−0.019–0.0273). Significant non-inferiority was achieved (upper limit: 0.0275) Ischemic stroke, SE, procedure-related events requiring major intervention in the first 7 days and all-cause death: Intervention group 2.2% (upper limit 95% CI 2.652%) Significant non-inferiority was achieved (upper limit: 2.67)
PRAGUE-17 (2020)	LAAO device (*n* = 201; amulet, Watchman o Watchman-FLX) versus DOAC non-inferiority (*n* = 201)	Follow-up: 20.8 ± 10.8 months Stroke or TIA, SE, clinically significant bleeding, significant periprocedural or device-related complications or CV death: LAAO 10.99% versus Control Group 13.42%; (*p*-value 0.004 for non-inferiority) Procedure or device-related complications occurred in only 9 patients (4.5%)

LAAO: left atrial appendage occlusion; OAC: oral anticoagulation; DOAC: direct oral anticoagulation; SE: systemic embolism; CV: cardiovascular; TIA: transient ischemic attack; RR: risk ratio; CI: confidence interval; HR: hazard ratio.

**Table 4:** Main procedural complications.

	PROTECT-AF (*n* = 463)	Prevail (*n* = 269)	PRAGUE-17 (*n* = 201)	Ewolution (*n* = 1021)	Post-FDA Approval (*n* = 3822)
Pericardial tamponade	22 (4.8%)	5 (1.9%)	2 (1%)	3 (0.3%)	39 (1%)
Vascular complications	-	1 (0.4%)	2 (1%)	5 (0.5%)	-
Major bleeding	22 (4.8%)	1 (0.4%)	2 (1%)	5 (0.5%)	-
Procedure-related stroke	5 (1.1%)	1 (0.4%)	-	1 (0.1%)	3 (0.08%)
Device embolization	3 (0.6%)	3 (0.7%)	1 (0.5%)	1 (0.1%)	9 (0.24%)
Procedure-related death	0 (0%)	0 (0%)	2 (1%)	1 (0.1%)	3 (0.08%)

**Table 5:** Characteristics of less widely used devices for LAAO.

Device	Category	Advantage	Special Indication
WaveCrest	Plug-based	Allows prerelease anytime reposition	Small LAA
Occlutech	Plug-based	Allows 180° rotation	Uncommon LAA anatomies
LAmbre LAA closure system	Disc-/lobe-based	Allows a complete recovery and repositioning	Small, multilobed, or chicken wing LAAs
Ultraseal	Disc-/lobe-based	High adaptability	Complexe LAA anatomies

LAA: left atrial appendage.

**Table 6:** Characteristics of ongoing randomized controlled trials.

Trial	Sample Size (N)	Intervention	Control
OPTION (NCT03795298)	1600	Percutaneous LAAO (Watchman FLX)	OAC
CHAMPION-AF (NCT04394546)	3000	Percutaneous LAAO (Watchman FLX)	DOAC
CATALYST (NCT04226547)	2650	Percutaneous LAAO (Amulet)	DOAC
OCCLUSION-AF (NCT03642509)	750	Percutaneous LAAO (Amulet or Watchman)	DOAC
ASAP-TOO (NCT02928497)	481	Percutaneous LAAO (Watchman FLX)	Single Antiplatelet Therapy or No Therapy (Control)
STROKE CLOSE (NCT02830152)	750	Percutaneous LAAO (Amulet)	OAC or NOAC, antiplatelet therapy (single or dual) or no therapy
CLOSURE-AF (NCT03463317)	1512	Percutaneous LAAO (Amulet or Watchman)	OAC (VKA or DOAC)
CLEARANCE (NCT04298723)	530	Percutaneous LAAO (Watchman FLX)	Standard of care (Best medical therapy for anticoagulation
COMPARE-LAAO (NCT04676880)	609	Percutaneous LAAO (Amulet or Watchman)	Antiplatelet or nothing
LeAAPS (NCT05478304)	6500	Surgical LAAO (AtriClip)	NO LAA Exclusion

LAAO: left atrial appendage occlusion; OAC: oral anticoagulation; DOAC: direct oral anticoagulation; SE: systemic embolism; CV: cardiovascular; TIA: transient ischemic attack; RR: risk ratio; CI: confidence interval; HR: hazard ratio.

## Data Availability

No new data were created or analyzed in this study. Data sharing is not applicable to this article.
